# Actions do not clearly impact auditory memory

**DOI:** 10.3389/fnhum.2023.1124784

**Published:** 2023-02-27

**Authors:** Marta Font-Alaminos, Nadia Paraskevoudi, Iria SanMiguel

**Affiliations:** ^1^Institut de Neurociències, Universitat de Barcelona, Barcelona, Spain; ^2^Brainlab-Cognitive Neuroscience Research Group, Departament de Psicologia Clinica i Psicobiologia, Universitat de Barcelona, Barcelona, Spain; ^3^Institut de Recerca Sant Joan de Déu, Esplugues de Llobregat, Spain

**Keywords:** action, production effect, self-generation effects, auditory memory, active learning

## Abstract

When memorizing a list of words, those that are read aloud are remembered better than those read silently, a phenomenon known as the production effect. There have been several attempts to understand the production effect, however, actions alone have not been examined as possible contributors. Stimuli that coincide with our own actions are processed differently compared to stimuli presented passively to us. These sensory response modulations may have an impact on how action-revolving inputs are stored in memory. In this study, we investigated whether actions could impact auditory memory. Participants listened to sounds presented either during or in between their actions. We measured electrophysiological responses to the sounds and tested participants’ memory of them. Results showed attenuation of sensory responses for action-coinciding sounds. However, we did not find a significant effect on memory performance. The absence of significant behavioral findings suggests that the production effect may be not dependent on the effects of actions *per se*. We conclude that action alone is not sufficient to improve memory performance, and thus elicit a production effect.

## Introduction

You have probably been told at least once to study aloud or while chewing gum to best prepare for an upcoming test. There are countless examples from daily life that suggest that actions could have an impact on memory performance. A related finding in scientific literature is the production effect. Several studies collectively have found that self-generated sounds (i.e., rehearsed piano melodies and spoken words) have better memory recall than their passively processed counterparts (Ekstrand et al., 1966; Hopkins and Edwards, 1972; Conway and Gathercole, 1987; Gathercole and Conway, 1988; MacDonald and MacLeod, 1998; MacLeod et al., 2010; Brown and Palmer, 2012; Mathias et al., 2015). The production effect’s memory mechanism(s) have been the subject of numerous theories, but one possibility that has not been considered is that movement in and of itself may contribute to this memory enhancement.

Stimuli generated by our own actions are processed differently than the inputs coming from external sources. Specifically, the most frequently reported finding has been sensory attenuation to self- compared to externally generated stimuli [see Horváth (2015), and Schröger et al. (2015), for a review of findings in the auditory modality]. In auditory research, most of the studies find attenuation of the N1 and P2 components of the event-related-potential (ERP). Typically, this sensory attenuation has been found for self-generated sounds (i.e., when the actions cause the sounds), however, several studies show attenuation even with mere action-sound coincidence (i.e., Hazemann et al., 1975; Makeig et al., 1996; Horváth et al., 2012; Horváth, 2013a,b). Indeed, movement has been shown to modulate sensory processing (Schafer and Marcus, 1973; Roy and Cullen, 2001; Hesse et al., 2010; Kelley and Bass, 2010; Requarth and Sawtell, 2011; Schneider et al., 2014; Chagnaud et al., 2015; Kim et al., 2015; Pyasik et al., 2018). One intriguing possibility is that movement may drive the activity of diffuse neuromodulatory systems such as the LC-NE system and thereby modulate responses in sensory cortices (Paraskevoudi and SanMiguel, 2023). Here, we ask whether movement, beyond sensory processing, may also modulate memory for concurrent sounds.

We hypothesize that the modulation of sensory responses during movement may have an impact on the memory encoding of concurrent stimuli, leading to an altered memory representation. Behaviorally, we expect that this can manifest as either an increased or decreased ability to remember the sounds depending on whether they coincided with an action or not during the encoding phase of a memory task. At the neural level, we expect to find indices of an altered memory representation. This may manifest as a modulation of sensory responses, that is, N1 and P2 attenuation, to the stimuli that coincided with movement during encoding when they are encountered again at retrieval. Alternatively, the modulation of sensory responses at encoding may in turn result in a modulation of the old/new effect, which consists of a more positive-going potential for correctly recognized old compared to new items and indexes the quality of conscious recollection (Sanquist et al., 1980; Warren, 1980, Wilding, 2000; Kayser et al., 2007; Rugg and Curran, 2007; Mecklinger et al., 2016; MacLeod and Donaldson, 2017).

## Materials and methods

### Participants

Twenty-two healthy subjects provided written consent and participated in the present study. The sample size was selected based on previous studies reporting robust self-generation effects (e.g., Horváth et al., 2012). Three participants were excluded from the analysis due to low signal-to-noise ratio on the electrophysiological data. Thus, the final sample consisted of 19 participants (6 males, mean age 22.74 years, range 18–29) that had a normal hearing, reported no history of psychiatric or neurological disease, and did not regularly consume psychoactive drugs nor in the 48 h before the experimental session. The study was approved by the Bioethics Committee of the University of Barcelona. Participants were monetarily compensated (10 euros per hour).

### Stimuli

We generated a total of 100 different environmental, natural, complex, and non-identifiable sounds. Samples were selected from the McDermott^1^ and the Adobe^2^ sound libraries. Non-identifiable sounds were selected to avoid, or at least minimize, semantic activation and instead focus the identification on the physical properties of the sounds. Sounds were sliced to a duration of 250 ms, ramped (0.01 s, exponential) and presented at 44.1 kHz, 16 bit and mono. The sound intensity was normalized across sound samples and adjusted to a comfortable hearing level. The 50 least identifiable sounds, according to an independent rating of 3 subjects, were used in the main experiment and the next 50 in the training.

### Experimental design

**FIGURE 1:**
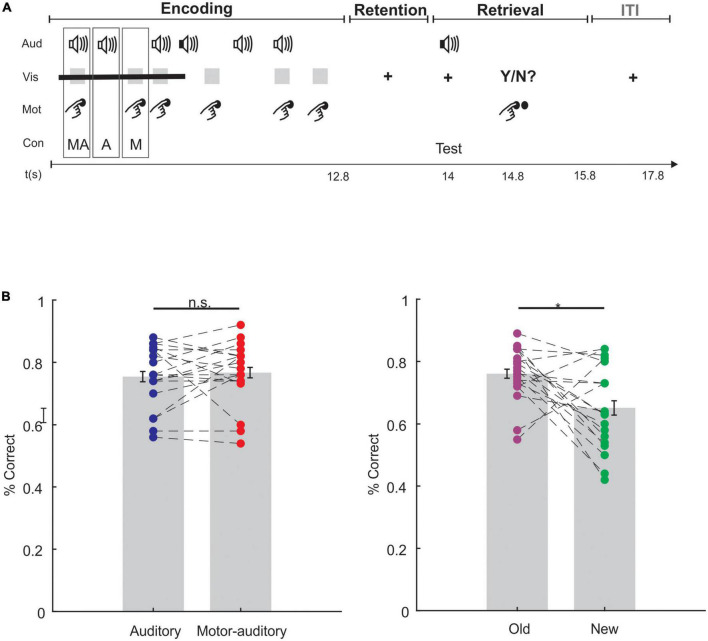
**(A)** Schematic description of a trial depicting the visual (Vis), auditory (Aud), and motor (Mot) occurrences taking place, and highlighting an example event for each condition (Con): motor-auditory (MA), auditory (A), and motor (M). Time in seconds, t(s): the timepoints mark the beginning of each phase of the trial. ITI, inter-trial interval. Finger used to generate sounds was the thumb. **(B)** Behavioral results. Bar plots with individual data points comparing the memory performance for the encoded as motor-auditory and encoded as auditory (left) and for the Old and New (right) sounds at retrieval. Individual data points are connected by a discontinuous line in each comparison. Error bars display the standard error of the mean (SEM). Asterisk denotes statistical significance.

The general design of the experiment was a Delayed-Match-to-Sample Task (DMTS), which consisted of 3 phases: encoding, retention, and retrieval. During the encoding phase, we exposed the subjects to auditory stimuli which they had to memorize. Half of the sounds were presented coinciding with a button press of the participant and constitute the Motor-auditory (MA) condition. The other half of the sounds were not related to any action of the participant and constitute the Auditory (A) condition. After a short retention period, we presented a test sound at retrieval. Participants responded whether the test sound was one of the sounds presented during the encoding and, thus, an old sound (Old condition) or a new sound (New condition, Figure 1A).

#### Encoding

At the beginning of each trial, the screen displayed 6 horizontally aligned and randomly spaced gray rectangles and a perpendicular, horizontal line that proceeded from left to right. Subjects pressed a button with their right thumb every time the line intersected a rectangle. Meanwhile, 6 sounds were presented which they had to memorize. On 50% of the presses, a sound was immediately presented after the press and the remaining sounds were presented between presses. Subjects were not told that some of the sounds will be generated by their actions. This resulted in 3 different event types: 3 × Motor condition (M): The subject pressed the button, but no sound was presented, 3 × A condition: A sound was presented without any action of the subject, 3 × MA condition: A sound was presented the moment the subject pressed the button. If subjects failed to press the button when indicated, an error message was presented, and the trial was aborted.

The total duration of the encoding phase was 12.8 s. The 9 encoding events occurred pseudo randomly within this time, with the following limitations: The event-to-event onset asynchrony varied randomly between 0.8 and 2.4 s. However, the minimum sound-to-sound onset asynchrony was 1.6 s. The last event occurred latest at 12 s, and it was always a sound event (MA or A). M events were always separated by at least one sound event.

#### Retention

After the encoding phase, a fixation cross was presented for 1.2 s. This was estimated as the minimum duration that would engage short term memory while minimizing echoic memory contributions (Crowder, 1976; Lu et al., 1992).

#### Retrieval

The test sound was presented 14 s after trial onset. A “Yes/No?” replaced the fixation cross on the screen 0.8 s after test sound onset, prompting participants to answer whether the test sound was old or new. The response window was 1 s. Once the participant responded, or after the response window ended, the question on the screen was replaced with a fixation cross until the onset of the next trial. The intertrial interval was 2 s.

Each of the 50 unique sounds used in the experiment served as the test sound in 4 trials. In these 4 trials, the sound sequences were composed of the same 6 encoding sounds and one test sound. However, two of these trials belonged to the Old condition, where the test sound was part of the encoding sequence, once presented coinciding with a button press (MA condition) and once presented without any action (A condition). The other two trials represented the New condition. These were identical to the Old condition, except that the test sound was replaced by another sound both at encoding and retrieval. The rest of the events of the trial (i.e., the other encoding sounds and the participant’s actions) were identical across the 4 trial versions generated for each unique sound.

The position of the test sound within the encoding sequence was chosen randomly for each unique sound. The positions could be from the second to the fifth, avoiding the first and last encoding sound positions to avoid primacy and recency effects (Mondor and Morin, 2004). However, to ensure that subjects did not learn to ignore those positions, 20 Catch-trials were added to the experiment with either position 1 or 6 for the encoding-test sound. The Catch-trials were not part of the analysis.

### Procedure

The experiment started with a progressive training where the participants learned how to perform the experiment in several short blocks of 5 trials each. First, they learned how to press the button on time whenever the line hit one of the rectangles, without auditory input. The word “error” appeared instantly on the screen every time they did not press the button on time. At the end of each block, feedback was presented on how many presses they missed and how many presses were not on time. Subsequently, auditory input was added, and subjects were instructed to perform the memory task. Here, the feedback screen at the end of each block also showed the “Misses” indicating unanswered questions or answers out of the required time window. Each part of the training was repeated until the subject could perform within minimal errors and misses.

After the successful training the experiment began which consisted of 22 blocks of 10 trials each, presented in randomized order. Total experimental time without pauses was 65 min. Subjects took short breaks between blocks to avoid fatigue.

### Apparatus

The experiment was performed in an electrically shielded chamber. The center of the screen was positioned at eye height, at 1.2 m. The EEG was recorded at a sampling rate of 500 Hz using Neuroscan 4.4 software *via* a SynAmps RT amplifier (NeuroScan, Compumedics). We used 64 Ag/AgCl electrodes inserted in a nylon cap (Quick-Cap; Compumedics) following the 10% extension of the International 10–20 system (Oostenveld and Praamstra, 2001). The EOG was recorded with NAS and one electrode under each eye (Schlögl et al., 2007). The reference was set at the tip of the nose and the AFz electrode served as the ground. Impedances were kept below 10 kΩ. Auditory stimuli were delivered binaurally *via* over-ear headphones (Sennheiser, HD 558). Participants’ button presses and responses were recorded with a silent response pad (Korg nanoPAD2). The setup was controlled and performed *via* MATLAB (The MathWorks)^3^ with the Psychophysics Toolbox (Brainard, 1997; Kleiner et al., 2007).

### Behavioral analysis

We calculated the percent of correct responses for sounds encoded as A and MA as well as for Old (both A and MA) and New sounds and performed a two-tailed paired samples *t*-test for each of the two comparisons (A-MA, Old-New). To complement our frequentist analysis, we conducted *post hoc* Bayesian *t*-tests to assess the evidence supporting a difference. We calculated the Bayes factor (*BF10*) for the alternative hypothesis (i.e., the difference of the means is not equal to zero), which was specified as a Cauchy prior distribution centered around 0 with a scaling factor of *r* = 0.707. The null hypothesis was specifically matched to an effect magnitude with a standardized effect size δ = 0 (Rouder et al., 2009). Data were viewed as moderate support for the alternative hypothesis if the *BF10* was larger than 3, whereas values close to 1 were considered only weak evidence and values below 0.3 were viewed as supporting the null hypothesis (Lee and Wagenmakers, 2013). Finally, to assess the bias in the responses we calculated sensitivity [as d’ = z(Hit) – z(False Alarm)] and criterion c = −0.5 * [z(Hit) + z(False Alarm)]; Roussel et al. (2013).

### EEG preprocessing and analysis

EEG analysis was performed with EEGLAB (Delorme and Makeig, 2004) and Eeprobe (ANT Neuro) was used for visualization. Data was high pass filtered at 0.5 Hz and non-stereotypical artifacts were manually rejected. We then applied Independent Component Analysis (ICA) decomposition using the binary version of the Infomax algorithm. After manual identification of the eye-movement artifactual components (Jung et al., 2000), the ICA weights of those components (mean components: 2.8) were removed from the raw data, already high pass filtered at 0.5 Hz. Subsequently, data was low pass filtered at 25 Hz and channels marked as broken at recording were interpolated.

Epochs were extracted from −0.1 to 0.5 s around the onset of each event of interest using the prestimulus period for baseline correction. At encoding epochs were defined for Auditory (eA) and Motor-auditory (eMA) sounds and Motor (eM) events; and at retrieval for encoded as Auditory (rA) and encoded as Motor-auditory (rMA) sounds. At retrieval, we also extracted epochs for correctly rejected New sounds, and for correctly recognized Old sounds, both as a whole and separately for those encoded as Auditory (rAcorrect) and Motor-auditory (rMAcorrect). Epochs with a voltage range exceeding 75 μV were rejected.

To test for the effects of actions on neural responses to sounds, we compared the auditory ERPs between MA and A events at encoding (eA vs. eMA) and between encoded as MA and encoded as A at retrieval (rA vs. rMA). At encoding, MA responses were corrected subtracting the ERP elicited by Motor events (eMA-eM) prior to this comparison. Both at encoding and retrieval, specifically, we tested for differences in the amplitude of the auditory N1 and P2 components at electrodes Cz and mastoids, and the N1 subcomponents Na and Tb at the collapsed electrodes T8 and T7, all identified and measured following SanMiguel et al. (2013). Given that P3 modulations have been reported (but not discussed) in previous work (e.g., Horváth et al., 2012), we decided to analyze P3 at encoding identified as the peak of the difference wave (A –[MA–M]) in the P3 window range based on previous work (e.g., Baess et al., 2008). At retrieval, the P3 component window served to test the old/new effect comparing responses between the correct New and correct Old (as a whole and separately for rAcorrect and rMAcorrect). We compared the mean amplitude of the components of interest in the identified time-windows at each electrode with two-tailed paired samples *t*-tests (Cz, Pz, collapsed mastoids and temporal electrodes) and with the *BF10* for consistency with the behavioral analysis.

## Results

### Behavioral

The overall memory performance was 70.57% (SD: 7.23). Accuracy for Old sounds did not differ based on how they were encoded [*t*(18) = −0.578, *p* = 0.571, *d* = −0.129, *BF*_10_ = 0.276; Figure 1B, left; see Table 1]. However, participants were better at recognizing old sounds than correctly rejecting new sounds [*t*(18) = 2.716, *p* = 0.014, *d* = 0.963, *BF*_10_ = 3.901; Figure 1B, right].

**TABLE 1:** Mean amplitudes and standard deviations of the results.

**Behavioral**					
			**% correct**	**D-prime**	**Criterion**
		**Condition**		**Mean (SD)**	
		A	0.75 (0.10)	1.26 (0.37)	−0.09 (0.27)
		MA	0.77 (0.10)	1.30 (0.44)	−0.11 (0.25)
		Old	0.76 (0.09)	1.27 (0.37)	−0.10 (0.25)
		New	0.65 (0.14)	1.27 (0.39)	0.22 (0.28)
**Electrophysiological**					
		**Encoding**		**Retrieval**	
**ERPs**	**Electrodes**	**Condition**	**Mean (SD)**	**Condition**	**Mean (SD)**
N1	Cz	A	−4.03 (1.68)	rA	−4.85 (2.61)
		MA	−3.46 (1.45)	rMA	−4.44 (2.06)
	Mastoids	A	0.39 (0.89)	rA	0.52 (1.07)
		MA	0.40 (0.94)	rMA	−0.07 (1.29)
Na	Temporal	A	−0.71 (1.07)	rA	−0.74 (1.08)
		MA	−0.87 (0.86)	rMA	−0.58 (1.31)
Tb	Temporal	A	−1.69 (1.02)	rA	−2.12 (1.65)
		MA	−1.09 (0.91)	rMA	−1.93 (1.25)
P2	Cz	A	3.04 (1.75)	rA	1.73 (2.54)
		MA	1.43 (1.18)	rMA	1.94 (2.18)
	Mastoids	A	−0.63 (0.89)	rA	−0.91 (1.18)
		MA	−0.44 (0.71)	rMA	−1.17 (1.22)
P3	Pz	A	0.02 (0.88)	rAcorrect	2.27 (2.97)
		MA	0.64 (0.91)	rMAcorrect	2.48 (2.35)
				Old	2.40 (2.45)
				New	0.88 (2.23)

D-prime did not differ between Old and New [*t*(18) = 0.164, *p* = 0.872, *d* = 0.008, *BF*_10_ = 0.240] nor between the A and MA conditions [*t*(18) = 0.621, *p* = 0.543, *d* = 0.112, *BF*_10_ = 0.282]. The Criterion measure differed between the Old and New [*t*(18) = −2.645, *p* = 0.016, *d* = −1.191, *BF*_10_ = 3.450]. However, it was similar for the A and MA conditions [*t*(18) = −0.621, *p* = 0.543, *d* = 0.086, *BF*_10_ = 0.282]. This reflects a more conservative strategy when judging new stimuli, however, the presence of an action does not affect the judgment strategy of old stimuli.

### Electrophysiological

#### Encoding

**FIGURE 2:**
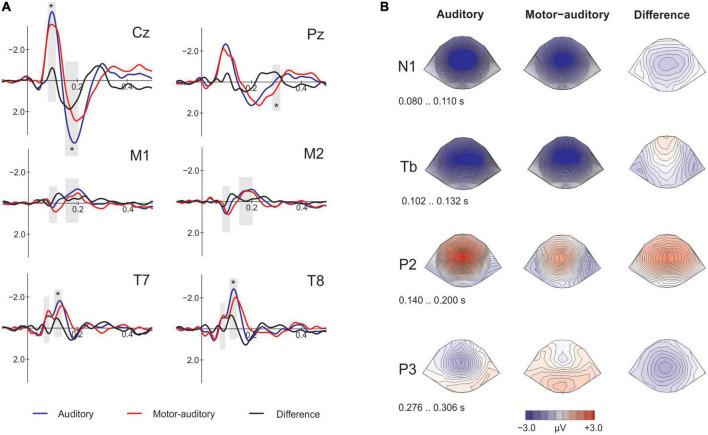
Electrophysiological results comparing the auditory and motor-auditory (motor corrected) stimuli at encoding. **(A)** Event-related-potentials (ERPs) on the analyzed electrodes. At Cz, M1 and M2 the analyzed components are N1 and P2, at T7 and T8 the N1 subcomponents Na and Tb, and at Pz the P3 component. The gray shading marks the time windows of the amplitude analysis. Asterisks mark significance. **(B)** Topographical plots of each component of interest.

To assess the effect of action on sensory responses, we contrasted the ERPs for the A and the motor corrected MA conditions (eA vs. eMA-eM; Figure 2). First, we identified the time-windows for the components N1 (80–110 ms) and P2 (140–200 ms) at the Cz electrode and at the mastoids, the N1 subcomponents Na (74–94 ms) and Tb (102–132 ms) at T7 and T8, and the P3 at Pz (276–306 ms). The analysis of the mean amplitudes (see Table 1) of the selected time-windows revealed a significant attenuation at Cz of N1 [*t*(18) = −2.452, *p* = 0.025, *d* = −0.56, *BF*_10_ = 2.487] and P2 [*t*(18) = 5.993, *p* < 0.001, *d* = 1.37, *BF*_10_ = 1957.803] for the MA condition. At the mastoids there were no differences on N1 [*t*(18) = −0.126, *p* = 0.901, *d* = −0.012, *BF*_10_ = 0.239] nor P2 [*t*(18) = −1.625, *p* = 0.122, *d* = −0.235, *BF*_10_ = 0.723] between conditions. Examining the temporal electrodes we found a significant attenuation of Tb for the MA condition [*t*(18) = −3.313, *p* = 0.004, *d* = −0.617, *BF*_10_ = 11.50], and no significant effects for Na [*t*(18) = 1.090, *p* = 0.290, *d* = 0.165, *BF*_10_ = 0.399]. At Pz, the P3 component revealed larger amplitudes for the MA condition [*t*(18) = −3.934, *p* = 0.001, *d* = −0.690, *BF*_10_ = 37.888].

#### Retrieval

**FIGURE 3:**
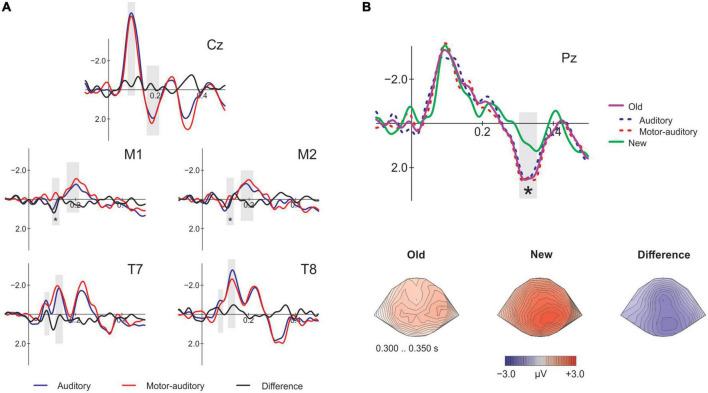
Electrophysiological results at retrieval. **(A)** Event-related-potentials (ERPs) comparing the encoded as auditory and motor-auditory sounds, passively presented at retrieval on the analyzed electrodes. At Cz, M1 and M2 the analyzed components are N1 and P2, at T7 and T8 the N1 subcomponents Na and Tb. The gray shading marks the time windows of the amplitude analysis. Asterisks mark significance. **(B)** Top figure: ERPs at Pz comparing the old and the new conditions. Auditory and motor-auditory conditions are displayed here for visualization purposes. Bottom figure: topographical plots in the P3 time-window showing the distribution of the old/new effect.

First, we assessed whether the source of the stimuli at encoding had an effect when presenting passively the same stimuli at retrieval by comparing the Old of the A and MA conditions (rA vs. rMA; Figure 3A). Then, we analyzed whether the old/new effect was modulated by the action effect comparing the correct Old for both A and MA with the correct New. To this end, we identified the time-windows for the components N1 (90–120 ms) and P2 (170–210 ms) at Cz and at the mastoids and the N1 subcomponents Na (60–90 ms) and Tb (120–150 ms) at T7 and T8. Additionally, to assess the memory old/new effect we identified the time-window for the P3 component at Pz (300–350 ms) for the correct responses at retrieval Old and New. The analysis of the mean amplitudes (see Table 1) of the selected time-windows for the contrast rA vs. rMA remained not significant for N1 [*t*(18) = −0.939, *p* = 0.360, *d* = −0.175, *BF*_10_ = 0.350] and P2 [*t*(18) = −0.433, *p* = 0.670, *d* = −0.088, *BF*_10_ = 0.258] at Cz. The P2 at the mastoids was in concordance with the findings on Cz [*t*(18) = 0.799, *p* = 0.435, *d* = 0.211, *BF*_10_ = 0.315], however, the N1 [*t*(18) = 2.671, *p* = 0.016, *d* = 0.500, *BF*_10_ = 3.604] revealed a significant enhancement for the sounds encoded as MA. Given that we did not obtain a significant N1 attenuation for the active condition at the Cz electrode, this mastoid attenuation should be treated with caution. As for the N1 subcomponents, we found no significant effects on Na [*t*(18) = −0.674, *p* = 0.509, *d* = −0.135, *BF*_10_ = 0.291] nor Tb [*t*(18) = −0.589, *p* = 0.563, *d* = −0.126, *BF*_10_ = 0.277]. Finally, the P3 old/new effect was significantly present at Pz between the Old and New [*t*(18) = 3.764, *p* = 0.001, *d* = 0.650, *BF*_10_ = 27.289], however, it did not differ between the rAcorrect and rMAcorrect condition [*t*(18) = −0.437, *p* = 0.667, *d* = −0.079, *BF*_10_ = 0.259; Figure 3B].

## Discussion

The goal of this study was to investigate whether actions alone could account for the production effect. Behavioral research has shown abundant evidence that sounds produced by oneself are better remembered than those just passively processed (Ekstrand et al., 1966; Hopkins and Edwards, 1972; Conway and Gathercole, 1987; Gathercole and Conway, 1988; MacDonald and MacLeod, 1998; MacLeod et al., 2010; Brown and Palmer, 2012; Mathias et al., 2015). However, since memory is a higher order process, it can be challenging to disentangle which lower-level processes are contributing to this complex effect. Normally, several co-occurring processes determine an outcome, thus, modulations of sensory responses could affect how action-revolving inputs are encoded in the memory stream.

In the auditory domain, self-generation effects refer to the attenuation of the sensory responses to a stimulus that has been produced by the same individual who is hearing the sound (SanMiguel et al., 2013; Saupe et al., 2013). Surprisingly, this effect persists even in the absence of contingency, that is, when the act performed does not actually generate the stimulus but occurs in the same time window (Horváth et al., 2012; Horváth, 2013a,b). Looking at the electrophysiological response during the encoding phase of our study we have replicated this result. The attenuation we measured for N1, Tb and P2 during encoding for sounds coinciding with actions is in line with well-established literature (Horváth, 2015; Schröger et al., 2015) and indicates the quality of our measurements. At encoding we also observed an increased P3 amplitude at Pz which may reflect the surprise of the sound that coincides with an action (Darriba et al., 2021), as in our experiment only half of the actions were accompanied by a sound (cf. Horváth et al., 2012; Paraskevoudi and SanMiguel, 2023). The surprising nature of the motor-auditory event could be obscuring the hypothetic memory encoding enhancement, and thus, result in the absence of memory improvement found for the motor-auditory sounds.

Could the action effects described at encoding contribute to the memory advantage observed in the production effect? We examined whether a non-contingent action-sound relationship affected memory performance on a task where old items could be either encoded coinciding with an action or not (i.e., motor-auditory and auditory sounds here). Our measurements showed evidence against an effect on auditory memory for action-coinciding stimuli. This indicates that actions alone do not facilitate the production effect. In line with our behavioral results, as the test sound was always externally generated, we could not find the typical self-generation effects at retrieval. However, our aim was to detect if there was any modulation in the sensory processing at retrieval dependent on the condition of the test sound at encoding.

Previous ERP research has reported the old/new effect, that is, correctly recognizing a previously heard sound elicits a more positive potential (onset at 300 ms) compared to hearing a new sound (Sanquist et al., 1980; Warren, 1980; Wilding, 2000; Kayser et al., 2007; Rugg and Curran, 2007; Mecklinger et al., 2016; MacLeod and Donaldson, 2017). In our study, this enhancement for the “Old” sounds at retrieval did not differ between previously encoded as motor-auditory and encoded as auditory sounds, indicating that the quality of recollection was also not affected by the presence of an action during encoding.

All in all, while we found a robust modulation of sound processing by actions during encoding, this did not seem to affect memory retrieval of these sounds, as we could not find any effects on the responses to the test sounds at retrieval. Hence, our data does not support a relationship between unspecific action effects of the coincidence of a sound with an action and memory accuracy. The null effect at retrieval could be related to the specific conditions of our experiment. We did not have sufficient trials to perform a remembered vs. forgotten analysis that could reveal the slight differences in performance that a coincidental action could be mediating. Interestingly, the sole study to date that tried to relate the memory advantage present on the production effect to the modulatory effects of motor activity surrounding auditory stimuli revealed worse memory performance to sounds coinciding with actions (Paraskevoudi and SanMiguel, 2023). One apparently minor difference between this and the former study is the type of question at retrieval. Both the yes/no and two-alternative forced-choice (2AFC) are formats often utilized in the recognition memory literature. In the yes/no format, used in the present study, the target stimulus was presented for a decision in isolation. This is known to require higher memory strength than the decision making between two stimuli (Jang et al., 2009). It could be possible that in Paraskevoudi and SanMiguel (2023) the 2AFC’s inherently greater performance made it easier to uncover the subtler differences between the two research conditions.

The absence of significant behavioral findings suggests that the production effect is not dependent on the presence of an action *per se*. We considered examining coincidental action was a logical first step to elucidate the role of action in the production effect. However, as we have evidenced, the surprise surrounding a coincidental action could be masking a co-occurrent memory enhancement. Future research with fully contingent paradigms will help clarify if there could be a memory advantage. We conclude the presence of an action alone is not sufficient to enhance auditory memory on a behavioral level and elicit a production effect.

## Data availability statement

The raw data supporting the conclusions of this article will be made available by the authors, without undue reservation.

## Ethics statement

The studies involving human participants were reviewed and approved by Bioethics Committee, University of Barcelona. The patients/participants provided their written informed consent to participate in this study.

## Author contributions

MF-A collected, analyzed the data, and wrote the first draft of the manuscript. NP and IS wrote sections of the manuscript. All authors contributed to conception and design of the study, manuscript revision, read, and approved the submitted version.

## Footnotes

1
http://mcdermottlab.mit.edu/svnh/Natural-Sound/Stimuli.html

2
https://www.adobe.com/products/audition/offers/AdobeAuditionDLCSFX.html

3
www.mathworks.com
